# Dynamics of Mutant Cells in Hierarchical Organized Tissues

**DOI:** 10.1371/journal.pcbi.1002290

**Published:** 2011-12-01

**Authors:** Benjamin Werner, David Dingli, Tom Lenaerts, Jorge M. Pacheco, Arne Traulsen

**Affiliations:** 1 Evolutionary Theory Group, Max-Planck-Institute for Evolutionary Biology, Plön, Germany; 2 Division of Hematology, Mayo Clinic, College of Medicine, Rochester, Minnesota, United States of America; 3 MLG, Département d'Informatique, Université Libre de Bruxelles, Brussels, Belgium; 4 AI-LAB Computer Science Department, Vrije Universiteit Brussel, Brussels, Belgium; 5 Departmento de Matemática e Aplicacões, Universidade do Minho, Braga, Portugal; 6 ATP-group, CMAF, Lisboa, Portugal; University of California-Irvine, United States of America

## Abstract

Most tissues in multicellular organisms are maintained by continuous cell renewal processes. However, high turnover of many cells implies a large number of error-prone cell divisions. Hierarchical organized tissue structures with stem cell driven cell differentiation provide one way to prevent the accumulation of mutations, because only few stem cells are long lived. We investigate the deterministic dynamics of cells in such a hierarchical multi compartment model, where each compartment represents a certain stage of cell differentiation. The dynamics of the interacting system is described by ordinary differential equations coupled across compartments. We present analytical solutions for these equations, calculate the corresponding extinction times and compare our results to individual based stochastic simulations. Our general compartment structure can be applied to different tissues, as for example hematopoiesis, the epidermis, or colonic crypts. The solutions provide a description of the average time development of stem cell and non stem cell driven mutants and can be used to illustrate general and specific features of the dynamics of mutant cells in such hierarchically structured populations. We illustrate one possible application of this approach by discussing the origin and dynamics of PIG-A mutant clones that are found in the bloodstream of virtually every healthy adult human. From this it is apparent, that not only the occurrence of a mutant but also the compartment of origin is of importance.

## Author Summary

We investigate the average stem cell driven dynamics of cell counts in an abstract multi compartment model. Within this framework one can represent different tissue structures, as for example hematopoiesis, the skin or the colonic crypt. Our analysis is based on an individual cell model in which cells can differentiate, reproduce or die. We give closed solutions to the corresponding system of coupled differential equations, that describe the average dynamics of all cell types. There are three cases of interest: (i) Mutations at the stem cell level, (ii) Mutations in downstream compartments associated with more mature, non stem cell types, (iii) Mutations in downstream compartments with cells acquiring stem cell like properties. The average dynamics shows for (i) and (iii) an increase of mutants towards an equilibrium, in case (ii) the average mutant cell count goes through a maximum, but mutants die out in the long run. We calculate the corresponding extinction times for every compartment. We discuss applications to hematopoietic diseases such as, PIG-A mutant cells or the classic oncogene BCR-ABL. Although the abstract model is a simplified sketch of cell differentiation, it is capable of describing many aspects of a wide variety of such tissues and associated diseases.

## Introduction

**Figure 1:**
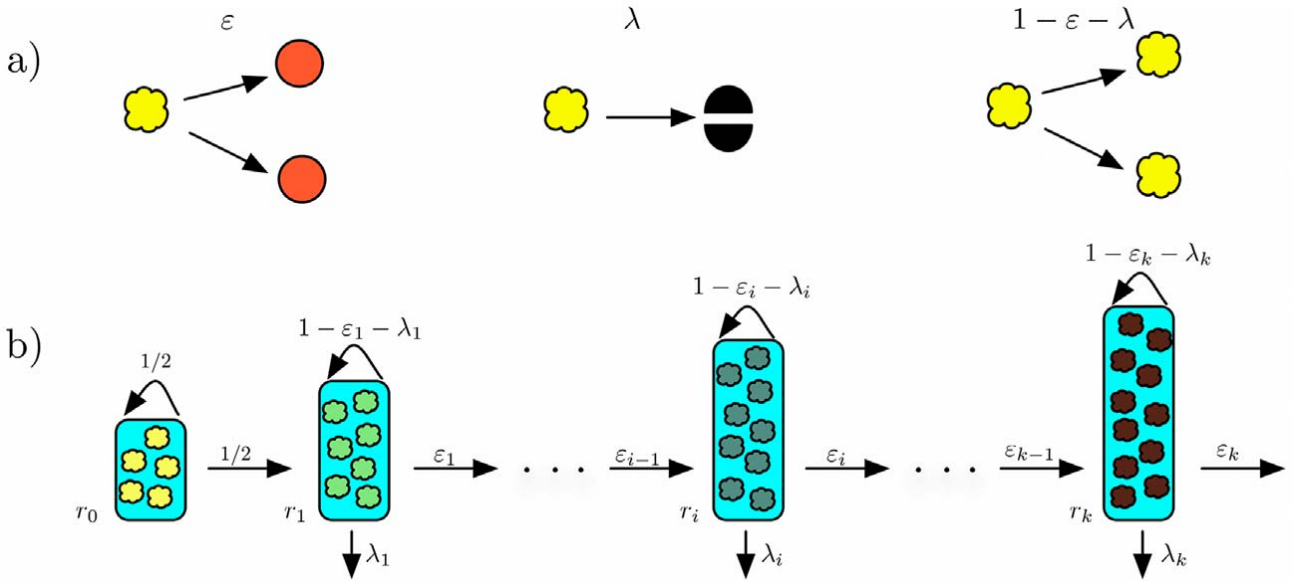
Cell proliferation and compartment structure. a) We consider three possible events during the cell division of a non-stem cell. Cells can differentiate, die, or reproduce. This happens with probabilities 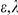, and 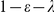, respectively. b) Compartment structure: The first compartment represents stem cells dividing asymmetric. These stem cells replicate with a rate 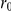 and their number 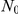 is constant. Cells in a non stem cell compartment 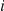 replicate with a rate 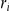. They will differentiate into the next compartment with probability 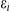, die with probability 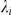 or produce cell types of compartment 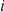 with probability 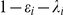.

Many tissues have a hierarchical multi compartment structure in which each compartment represents a cell type at a certain stage of differentiation. This architecture has been well described for hematopoiesis [1], [2] and epidermal cell turnover in the skin [3], [4] or in the colonic crypt [5]. At the root of this process are the tissue specific stem cells that have the capacity to differentiate into more specialized cells [6]. Each cell undergoes a series of cell divisions and differentiation steps until the whole diversity of the tissue is obtained [1], [2], [7]–[11]. The model presented here closely follows this concept. We introduce in total 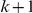 compartments, where each compartment 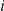 represents a certain stage of cell differentiation with 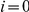 representing the stem cell pool. Each cell in a compartment 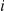 replicates at a rate 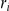. If a cell in a non stem cell compartment 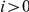 replicates, it can undergo three different processes: With probability 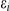, it divides into two more differentiated cells that migrate into the adjacent downstream compartment 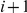. With probability 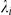, the cell dies. With probability 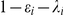, it divides into two cells that retain the properties of their parent cell and therefore remain in the same compartment 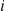 (self-renewal), as shown in Fig. 1. Thus in compartment 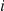, the number of cells 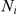 is increased by influx from the adjacent upstream compartment 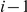 and self-renewal within compartment 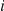, and decreased by cell death in compartment 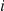 and cell differentiation into the adjacent downstream compartment 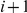. One could also allow asymmetric cell divisions in non stem cell compartments. But the average dynamics in this case can be captured by modifying the differentiation probabilities 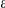. Thus, this case is implicitly included in our model. In the following, we shall assume a constant number of stem cells 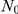, following [1], [12]. This can be achieved via asymmetric cell division [13], [14]. However, one can also assume a process at the stem cell level in which cell differentiation, cell death and self renewal are balanced such that the average number of cells remains constant, i.e. 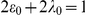. For immortal stem cells, 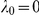, this means 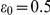. However, for our purpose details of the dynamics in the stem cell compartment are not relevant, as long as the number of stem cells is constant.

This model does neglect several aspects that may have an impact on the dynamics of the system under consideration, such as biochemical feedback or spatial population structure [15]–[19]. However, due to the generality, our model can be seen as a benchmark and thus allows to infer when such aspects are of importance and when they can be ignored by a comparison between the different model classes.

One special case of our framework is the model of hematopoiesis discussed in [1]. There, cell death is neglected, 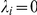 for all 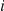. Furthermore, an exponentially increasing proliferation rate 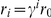 and a constant differentiation probability 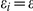 are assumed for all non stem cell compartments. In this work, we relax these conditions and therefore our analytical arguments hold for general values of 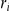, 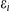 and 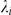 in each compartment and thus for a wide class of related models and different tissues.

## Methods

### Mathematical model

The individual cell model is based on a finite number of cells that divide and differentiate with certain probabilities. Thus, it is a stochastic process [20] and fits the current view of the stochastic nature of such cell differentiation processes [21]. However, the average cell numbers can be captured by a system of coupled differential equations that is deterministic. These equations follow from a proper counting of incoming and outgoing cells within each compartment 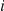.

Let us assume that the number of stem cells 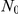 is constant, following [1], [12]. The number of cells in the first non stem cell compartment 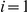 increases by influx from the stem cell pool at a rate 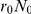 and due to self renewal at a rate 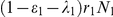. In addition, the average number of cells in the compartment 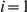 is lowered by cell differentiation into the next compartment 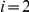 at rate 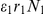. Cell death in compartment 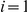 occurs at rate 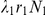. The dynamics in all other compartments is the same, except that the number of cells 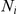 in the compartment 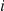 increases due to influx from the adjacent upstream compartment at rate 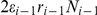. Self renewal occurs at rate 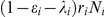. 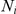 decreases due to cell death at rate 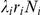 and cell differentiation at rate 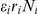. Combining these terms and assuming in total 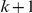 compartments, we obtain a system of coupled differential equations
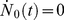
(1a)

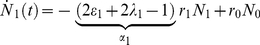
(1b)

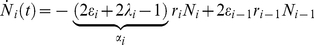
(1c)where 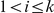 and the dots denote derivatives with respect to the time 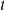. From now on, we use the abbreviation 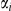 to denote the difference between the loss from compartment 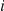 due to differentiation and cell death and the gain from self renewal. Thus, 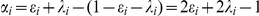. Typically, we will have 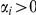, and this net loss of cells will be compensated from the influx of cells from the upstream compartment.

### Stochastic simulation

The simulations presented in this paper are individual based stochastic simulations. We implement all elements of the first 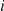 compartments separately, thus we are able to record the dynamics of every single cell. Every cell division is called an event. We use a standard Gillespie algorithm [22] to determine in which compartment the next event takes place. After the compartment is determined, one cell in this compartment is chosen to divide proportional to the reproduction rate. The outcome of this event is determined by the cell death and differentiation probabilities 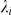 and 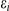. The dynamics in the stem cell compartment is different: in our realization stem cells are allowed to divide asymmetrically only, thus we keep the number of stem cells constant. One could implement a Moran process on the stem cell level also and therefore allow dynamics on the stem cells [14]. However this would not change the aspects we look at in this paper. The number of stem cell events determine the time scale. We define 1 time unit as 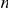 stem cell events. For example, in the hematopoietic system of a healthy adult human we assume that there are approximately 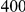 stem cell divisions a year.

## Results

### Stem cell driven dynamics

The equilibrium of the process is obtained from setting the left hand side of our system of differential equations to zero. Biologically, this corresponds to tissue homeostasis. In this case, we have
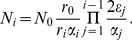
(2)


Next, we turn to the process of filling empty compartments by a continuous influx from the stem cell pool. Because we do not consider interactions between different cell clones in our differential equations, this corresponds also to the dynamics of a mutation arising in the stem cell pool. Thus, we choose the initial condition
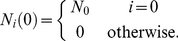
(3)The differential equation (1b) for the compartment 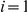 is an inhomogeneous linear differential equation of first order and can be solved by methods such as the variation of parameters. Assuming initial condition (3), one obtains the solution for compartment 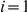


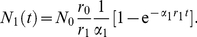
(4)Because the differential equation for compartment 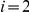 depends on 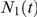 and 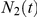 only, one can insert (4) into (1c) for 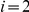 and solve the resulting inhomogeneous equation through variation of parameters again,
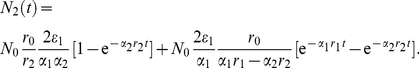
(5)Continuing with this procedure one can find the general pattern, which leads to a solution for general 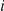, 
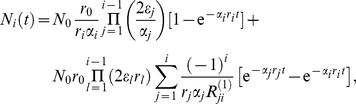
(6)where we have introduced 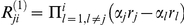 to shorten our notation. Equation (6) allows any choice of 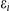, 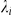 and 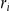. Within the basic model assumptions depicted in Fig. 1, this represents the most general case. All thinkable stem cell driven effects can now be described and followed in detail, as for example any change in the equilibrium compartment sizes or any change of cell division properties during cell differentiation. Compartments are continuously filled with cells until they reach the equilibrium described above. This can easily be deduced from (6), because all terms involving decaying exponential functions in time will ultimately be irrelevant for the cell counts.

If we choose (i) an exponentially increasing proliferation rate 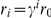, (ii) constant differentiation probability 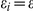 and (iii) constant cell death 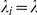 for each non stem cell compartment 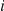, solution (6) simplifies to 
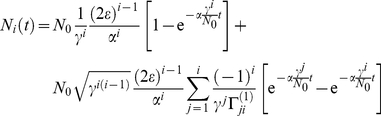
(7) with 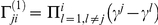 as a short cut.

**Figure 2:**
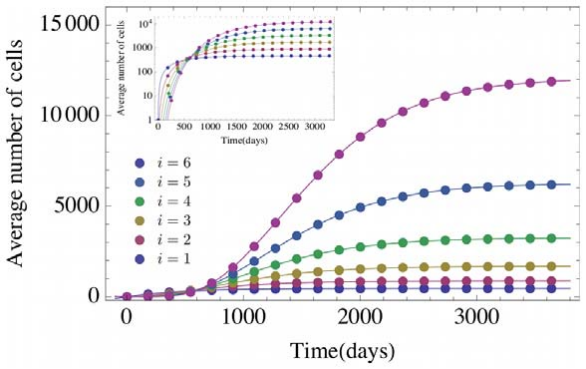
Deterministic dynamics of hematopoiesis modeled as a hierarchical multi compartment process. The colored symbols are averages of an individual based stochastic simulation with 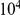 realizations and corresponding standard deviations. Missing error bars are smaller than the point size. The colored lines are our analytical solution (7) for the first six non stem-cell compartments 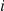. Parameters are based on the model of hematopoiesis in [26] (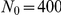, 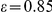, 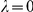, and 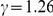, stem cells divides approximately once per year). The stem cell division rate fixes the time scale. Compartments are continuously filled with cells until they reach the equilibrium.

In Fig. 2, equation (7) is compared to averages obtained from an individual based stochastic simulation. Note that 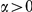 is required to maintain an equilibrium. In this case we have 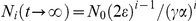, the cell count in a compartment 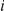 under equilibrium conditions. This is in agreement with former results [23]. While we focus on the biologically relevant case of 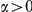, we can also consider more general values of 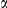. For 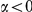, the compartment produces more cells than it loses even in the absence of cell influx from upstream. Thus, the number of cells would grow exponentially according to equation (7) in each non stem cell compartment. For 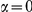 the gain of cells due to self renewal and the loss of cells due to differentiation and cell death in a compartment is equal. Thus the number of cells are not changed by processes in the compartment, despite a continuous output of cells into the next downstream compartment. The case 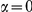 can be solved directly from Eqs. (1a)–(1c), which gives 
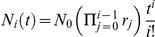
.

Solution (6) describes the deterministic process of filling empty compartments within hierarchical organized tissue structures, as can occur during wound healing, recovery from hematopoietic stem cell transplantation [18] or of in vitro experiments with fetal liver cells [24]. However, it can also be viewed as the dynamics of a mutant clone arising from a single cell in the stem cell pool, 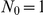. Thus, it is also possible to describe the average time development of diseases caused by mutations at the stem cell level such as the chronic myeloid neoplasms. Again, because we assume there is no interaction between normal cells and mutated cells, the dynamics of mutated cells proceeds independently, albeit with different differentiation parameters.

### Non stem cell driven dynamics

Next, we turn to mutations occurring downstream of the stem cell compartment. The occurrence of a mutation in a non stem cell compartment is more likely than a mutation in the stem cell pool due to the higher numbers and proliferation rates of non stem cells. The dynamics of such a mutant is not driven by the stem cell pool and thus is not described by the solution form above, equation (6). However, the compartment structure is unchanged and thus the dynamics of such mutants is also described by equations (1a)–(1c), but with altered initial conditions. Assuming there is a mutation in compartment 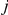, the initial condition is
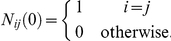
Here 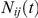 represents the number of mutant cells in compartment 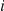 at time 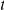, whereas the mutation occurred in compartment 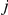 at time 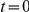. According to this initial condition, the system of coupled differential equations (1a)–(1c) turns into a homogenous system and the dynamics of mutant cells is independent from the first 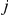 equations, see Text S1 for details. Using the same tools as above, we obtain the general solution
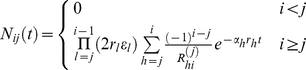
(8)with 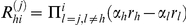. Note that this equation describes the dynamics of chronic myeloid leukemia clones in [10] analytically and reduces to the solution in [17] in a special case. If, as in [1], we assume (i) an exponentially increasing proliferation rate, (ii) a constant differentiation probability, (iii) constant cell death across all compartments, then the solution simplifies to
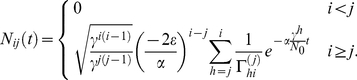
(9)


**Figure 3:**
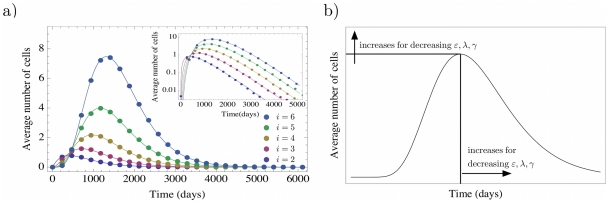
Deterministic dynamics of non stem cell driven mutants in a hierarchical multi compartment process. a) A single mutant occurred in the compartment 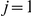. Shown are the number of mutants in the downstream compartments 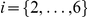. Lines are due to equation (9), the colored dots are due to an individual based stochastic simulation. The corresponding parameters have been chosen from a model of hematopoiesis [26] (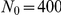, 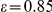, 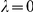 and 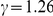). Averages are over 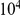 realizations. In the long run, mutants will always be washed out, because of the missing input from the stem cell pool. However, this process can take a very long time, in our example it would be of the order of 10 years until the average number of mutant cells becomes smaller than one in compartment 6. But it will take significantly longer until all cells in downstream compartments are washed out. b) Parameter dependence of the maximum. For decreasing 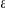, 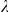 and 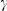 the maximum number of average cells increases, but the time to reach this maximum increases. The more differentiated the cell of the origin of the mutation, the lower the maximum number of cells and the quicker this maximum is reached.

**Figure 4:**
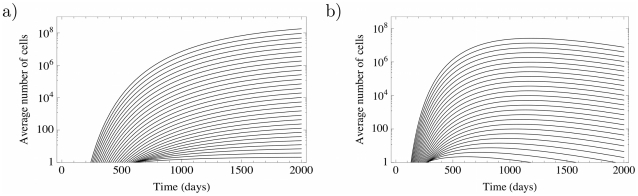
Average cell number for first 31 compartments. a) Average number of mutants in the first 31 compartments of a hematopoiesis model (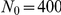, 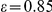, 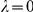 and 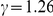) driven by a single stem cell mutation, cf. equation (7). The number of mutants increases exponentially per compartment and approaches an equilibrium. b) The same dynamics driven by a single mutant in the third non stem cell compartment 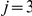, due to equation (9). The average mutant cell count reaches a maximum, but vanishes in the long run, because of the missing influx from the stem cell compartment. However, it will be difficult to distinguish between the two cases in the initial phase of the disease (here 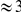 years).

Fig. 3 a) shows the dynamics of mutants in the first compartments, when the mutation arises in compartment 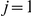. Note that for the most biologically plausible case 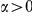, for large 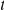 the exponential functions in (8) vanish. Thus the mutants will be washed out from the non stem cell compartments. Thus, the absence of mutants is a stable state of such hierarchical compartment structures. However, this equilibrium may not be of any biological or medical relevance, since the time to get rid of the last mutant cells of the clone may be longer than the normal expected lifetime of the healthy organism, cf. Fig. 4. Fig. 3 b) shows how the maximum of equation (9) and the time to reach it depends on 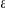, 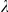, and 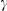. As the proliferation rate 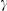 of the mutant population decreases, the size of the mutant population in downstream compartments increases, although it will take ‘longer’ for the population to reach high levels. A mutation that increases the net loss of cells 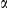 (either by increasing cell differentiation 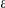 or cell death 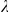) in a compartment lowers the number of mutants at maximum size of the clone in downstream compartments, which is also reached earlier. Note also that mutations occurring later in the cell differentiation process will lead to smaller maxima that vanish faster [25].

Based on equation (8), other mutant dynamics are also possible. If 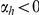 in a single compartment 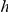, mutant counts diverge exponentially in all downstream compartments. If 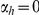 in a single compartment 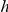 and 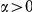 otherwise, in the long run mutants will reach an equilibrium in all downstream compartments, which is given by
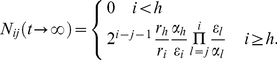
(10)This equilibrium is robust against variations of 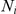 and thus is a stable fixed point. However a small change in 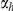 would lead either to extinction or the divergence of the mutant cell count. For a more detailed discussion, see Text S1. Initially, the difference between the dynamics of a clone arising from the stem cell compartment and an early non stem cell compartment is small, see Fig. 4.

### Mutant extinction times

**Figure 5:**
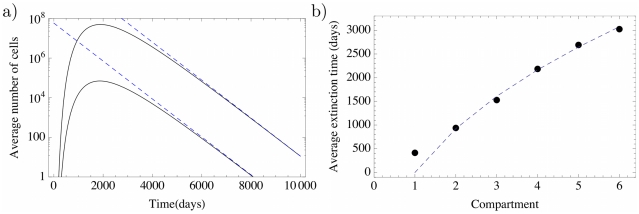
Extinction times. a) The black lines show the average mutant cell count based on equation (11) in the compartments 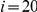 and 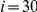, where the mutant occurred in the first non stem cell compartment 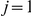. The dashed blue lines are given by equation (11), extinction occurs when the average cell count drops below 1. Again the parameters are chosen from a model of hematopoiesis as in Fig. 3 and 4. b) Average extinction time due to simulation (black dots) and due to equation (14) (dashed blue line). This approximation does not work for the mutant in the compartment of origin, where an alternative approach is necessary [23].

In the long run the average mutant cell count is given by the dynamics of the slowest decaying exponential function of equation (8). It is often natural to assume that this corresponds to the dynamics in the compartment of the mutant origin 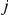. Thus, if we assume that 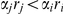 for all 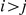 (as in the hematopoiesis model in [1]), in the long run mutants will die out at a rate
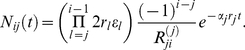
(11)This is shown in Fig. 5 a). For this special choice of parameters equation (11) becomes
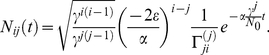
(12)Thus, the mutant cell count in the long run is given by a decaying exponential function. This enables us to calculate the average extinction time 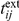 of mutants in the 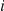-th compartment, if the mutation occurred in compartment 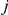,
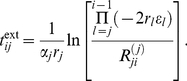
(13)If we assume a constant differentiation probability, constant cell death and an exponential increasing proliferation rate again this simplifies to
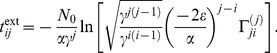
(14)In Fig. 5 b) we compare the extinction time due to equation (14) to simulation results. This approximation does not allow to calculate the extinction time of the mutant of the compartment of origin, but a more detailed consideration of this case can be found in [23].

A special case of interest is a mutation with 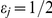 and 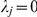. This results in a mutant cell that shows stem cell like properties in compartment 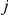 and non stem cell like properties in higher compartments. In this case the set of differential equations (1a)–(1c) becomes
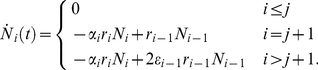
(15)Equation (15) transforms into equation (1a)–(1c) if one shifts the index 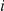 to 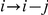. Thus we have to shift the index of the general solution (6) and find 
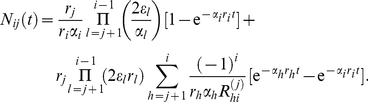
(16)Thus, the average dynamics of such a mutation is exactly as described above for a stem cell mutation. The averages of the simulation are described by equation (6) and (8), but a single run is still stochastic. The relative standard deviation of lower compartments 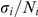 is of order 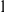, but decreases with increasing compartment number. Stochastic effects on the stem and the early progenitor cell level are important and can be crucial in a medical context [14], [26], but to understand some fundamental properties, the deterministic view seems to be sufficient.

### Example: Dynamics of PIG-A mutants

Here, we will utilize the model to illustrate the dynamics of a mutation that is seen in virtually every healthy human being. Sensitive flow cytometric analysis of circulating blood cells will identify a small clone that lacks expression of CD55 and CD59 (amongst others) [27]. CD55 and CD59 belong to a class of proteins that inhibit complement activation and their absence renders red blood cells sensitive to intravascular destruction. These proteins are normally displayed on the surface of cells since they are anchored to the plasma membrane via a glycosylphosphatidyl inositol (GPI) moiety. Synthesis of GPI requires a series of steps. The PIG-A gene encodes a protein that is an essential component of the complex responsible for the first step of GPI biosynthesis. Mutations in this gene can lead to a partial or complete deficiency of GPI synthesis resulting in low level or complete absence of such proteins from the cell surface, as for example the complement inhibitors CD55 and CD59 [28], [29]. Red blood cells lacking CD55 and CD59 are destroyed by complement, leading to hemolytic anemia. As a result, mutations in PIG-A can explain the phenotype of paroxysmal nocturnal hemoglobinuria (PNH), an acquired hematopoietic stem cell disorder characterized by anemia, hemoglobinuria and other manifestations [30]. A recent mathematical model suggests that a PIG-A mutation in a HSC is sufficient to explain the incidence and natural history of PNH [31]. However circulating blood cells with the PNH phenotype (due to a mutation in PIG-A) can be found in virtually every healthy adult human [27]. Such clones generally disappear with time. With this background, we will now apply the analytical solution (8), to assess extinction times of PIG-A mutants and compare these results to in vivo data derived from healthy adult humans.

The model parameters were fixed to represent hematopoeisis following [1]. In this approach, cell death is neglected, 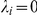 for all 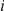, and an exponentially increasing proliferation rate 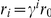 as well as a constant differentiation probability 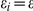 is assumed for all non stem cell compartments. Further, limited self-renewal is considered across many stages of differentiation, a prediction that is finding increasing support. For example, this was noted recently for cells at the proerythroblast stage, a cell type far removed from the stem cell or primitive progenitor cell pools [32]. Finally, the model parameters for human hematopoiesis become 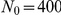, 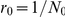, 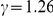 and 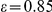. The number of cells per compartment increases exponentially and one needs 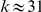 compartments [1] to ensure that in a healthy adult human, on average, the daily bone marrow output is of the order of 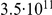 blood cells [33]. The same model can also be fitted to other mammals [34]. PIG-A mutants are considered to be neutral [35], supported by in vivo measurments [36], [37]. Thus we chose, as for normal cells, 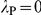 and 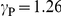 as mutant parameters. For fully neutral mutants, the clone would either be present for too much time or it would not reach the level observed in vivo. We explored various values of 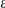 and found that a slightly lower differentiation probability 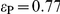 gave the best results. Note that this slight difference compared to healthy cells is still consistent with the experimental evidence.

**Figure 6:**
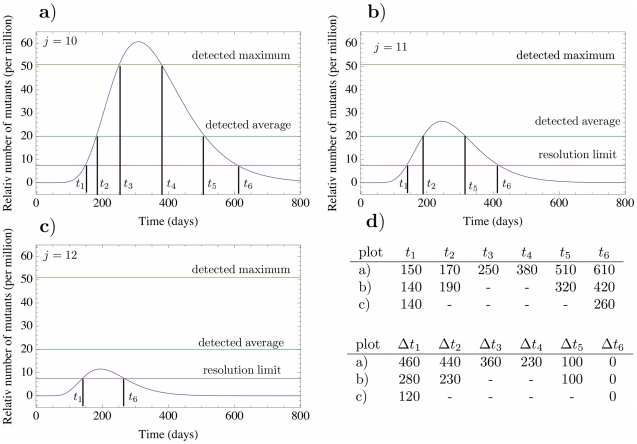
Dynamics of PIG-A mutants. Panels a) to c) show the number of PIG-A mutants per million healthy cells in compartment 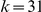, based on equation (8). The mutation of origin occurred at time 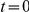 in compartment 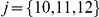. The parameters of the mutant cells are 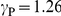, 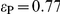 and 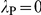 in all compartments. The horizontal lines correspond to the detected maximum, the detected average and the detection limit of PIG-A mutants observed in in vivo studies [27]. The upper table in panel d) shows the time (in days) of these events after the original mutation occurred at time 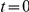 based on equation (8). The bottom table shows the corresponding extinction times (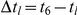). We predict an extinction time of 230 days for the measured maximum and 100 days for the average mutant cell count.

In Araten et al. [27] the blood of 19 healthy adult humans was sampled and tested for clones with PIG-A mutations. Mutants were found in every person ranging from 8 to 51 mutants per million (with an average of 21) normal blood cells. Blood samples from the same patients were taken at later times to determine survival of these clones. The lower limit of detection in [27] was approximately 7.5 mutant cells per million. The detected maximum of 51 mutants per million healthy cells decreased after 164 days and was undetectable after 192 days. Individuals with the average cell count of 21 PIG-A mutants per million still had the clone present after 65 days, but it was not detectable after 174 days. We need to determine the compartment 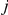, where a mutation in PIG-A occurred, such that the clone that arises would grow to reach the detection threshold and remain detectable for a time compatible with observations. Using equation (8), we record the dynamics of mutant cells in compartment 31 for different compartments of origin. In Fig. 6, the mutant cell count per healthy cells in compartment 31 is shown, where the mutation took place in a) compartment 10, b) compartment 11 and c) compartment 12. With these curves, one can predict extinction times for different origins of the mutation. Comparing the total size of the mutant population and the corresponding extinction times to values obtained in humans [27] allows to predict the compartments where the mutant clone originated.

In Fig. 6, we show the corresponding times from the mathematical model calculated from equation (8). The same figure also illustrates that the time of origin of the mutation can be much earlier than the detection time. For example, if the mutation occurred in compartment 10, we predict an extinction time of 230 days for the maximum of 51 mutants per million, when the initially sample was taken at time 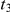, see figure 6 a). The extinction time for the average cell count is 100 days. However, it should be clear that such mutant cells will survive for significantly longer than what is detectable by technology due to issues of sensitivity. With this in mind, there is good agreement between what the model predicts and the results described in [27], since it is unlikely that the clones in all the individuals were either found as soon as they were detectable or when they were at their peak concentration. Thus what is relevant are (i) the distribution of times that these mutant cells remain in circulation and (ii) the size of the clones one observes. In this respect our model provides a very good approximation of the dynamics of such clones and is able to infer the cell of origin. If the mutation occurred in earlier compartments, the clone would be expected to expand to a higher cell count and will stay in the circulation for a longer time, but such clones are less likely to occur due to the lower number of progenitor cells and slower proliferation rate. Mutation events in higher compartments as 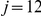 are more likely to happen but these mutants would not be detectable by most current clinical flow cytometry techniques due to the small size of such clones in compartment 31 although they may be detected perhaps with polymerase chain reaction technology. Thus, compartments 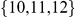 are the most likely compartments of the mutant origin for the cases described in [27]. Mutations arising in these compartments correspond to mutations in early progenitor cells. This agrees with the experimental results, since the PNH phenotype is present in several different cell lineages and thus has to occur early in the hematopoietic tree. Note that besides the compartment of origin, the only free parameter is the differentiation probability of mutated cells 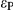. Here we assumed that these circulating mutant cells all originate from one founder cell. Two or more independent, simultaneous or contemporaneous clones in early compartments would be unlikely [23], [38]. If a second independent mutation occurs during the presence of an earlier mutant population, the total mutant cell number is the sum of both single populations, see Text S1 and Figure S1 for more details.

Moreover the hierarchical structure of hematopoiesis provides an explanation why almost all humans carrying PIG-A mutations do not have symptoms of PNH. Only mutations in the most ‘primitive’ compartments have an impact and only mutations in a HSC will lead to disease. In general, one can predict the dynamics for mutants with very different properties using equation (8). The compartment of the mutant origin can be inferred if one follows the mutant count by taking blood samples at regular intervals.

## Discussion

In this work, we presented closed analytical solutions for the deterministic dynamics of stem cell and non stem cell driven mutants in a multi compartment model of tissues such as hematopoiesis, the skin and the colon. This enables us to describe the dynamics of mutant cells in a general approach. We can predict the time development of a mutant depending on its origin and its specific proliferation properties. The process of cell differentiation is conceptually fairly well understood, but it is of course a challenge to estimate the various parameters in our model for real systems. Fortunately, very often, simplifying assumptions, e.g. exponentially increasing cell proliferation rates, can lead to insights [26]. However, our analytical solution allows us to incorporate more involved parameter dependencies, which could immediately be analyzed.

Let us turn to hematopoiesis to address some of the implications of our model because recent technological developments allow the detection of well known mutations in many otherwise healthy people. Perhaps the best examples are derived from blood disorders, since repeated blood sampling is a minimal invasive procedure and molecular probes for many blood disorders are available. The case of PIG-A mutant cells present in healthy humans has been analyzed extensively in an earlier section. There are several other specific examples [25].

- A mutation in the janus like kinase 2 where phenyalanine substitutes valine (JAK2V617F) is a common mutation in patients with chronic myeloid neoplasms. However, one can find this mutation in a substantial fraction of healthy adults (perhaps 0.2–0.4 percent) and with an even higher frequency (0.94 percent) in hospitalized patients who do not have an overt hematologic disorder [39], [40]. JAK2V617F is expected to give a survival and reproductive advantage to cells, and probably also enhances self renewal of progenitor cells. Knowing the dynamics of such clones, can lead to an understanding of the cell of origin in these patients as well as its impact on the fitness of mutant cells.
- Finally, the classic oncogene BCR-ABL [41] that is associated with chronic myeloid leukemia can be found in healthy adults [42]. In some of these individuals the mutant clone resolves while in others it persists but to our knowledge, none of the individuals in the cohort described have progressed to develop CML. There are various potential explanations for this observation including (i) non-stem cell origin of the mutant clone, (ii) stochastic extinction [14], (iii) immune response to the clone, (iv) additional mutations may be needed to lead to CML. Independent of the multitude of possibilities, it is safe to conclude that the cell of origin of a mutant is of importance and the impact of a mutation is cell context dependent. Our model can provide plausible explanations for the frequency and cell of origin of these mutations and perhaps why they do not lead to disease.
- We can also think of other mutations altering cell division properties. For instance, one can consider a mutation occurring in compartment 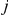 with 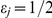, 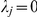 and normal properties in all the other upstream compartments. This would be the special case described by equation (16), and can be understood as a mutation that enables a cell to reacquire stem cell-like renewal capacities again. Such a behavior can explain the origin of various subtypes of acute leukemia as has been reported recently [43]–[45].

Our model provides a mathematical abstraction of hierarchically structured tissues and neglects many factors that can have an important impact on the dynamics, as for example spatial population structure or temporal changes of cell division properties, e.g. due to aging or injury. Nonetheless, the most important aspects of such tissue structures are captured by our model. It takes the form of ordinary differential equations that allows analytical solutions in many cases. An alternative would be a numerical solution, but such a solution has to be implemented for specific sets of parameters. We are convinced that our model can readily be applied to various hierarchical tissues and expect that general features of mutant dynamics will be conserved across different tissues.

## Supporting Information

Figure S1 **Example of three independent mutation events with equal properties.** Shown is the overlapping dynamics of three independent mutation events.
(PDF)

Text S1 **Details of mutant cell dynamics.** Discussion of mutant cell dynamics and overlapping mutation events.
(PDF)
