# Supplementary figures and images for: Dynamics of Mutant Cells in Hierarchical Organized Tissues
Source: PLoS Comput Biol. 2011 Dec 1;7(12):e1002290. doi: 10.1371/journal.pcbi.1002290 (PMC3228763; doi:10.1371/journal.pcbi.1002290)

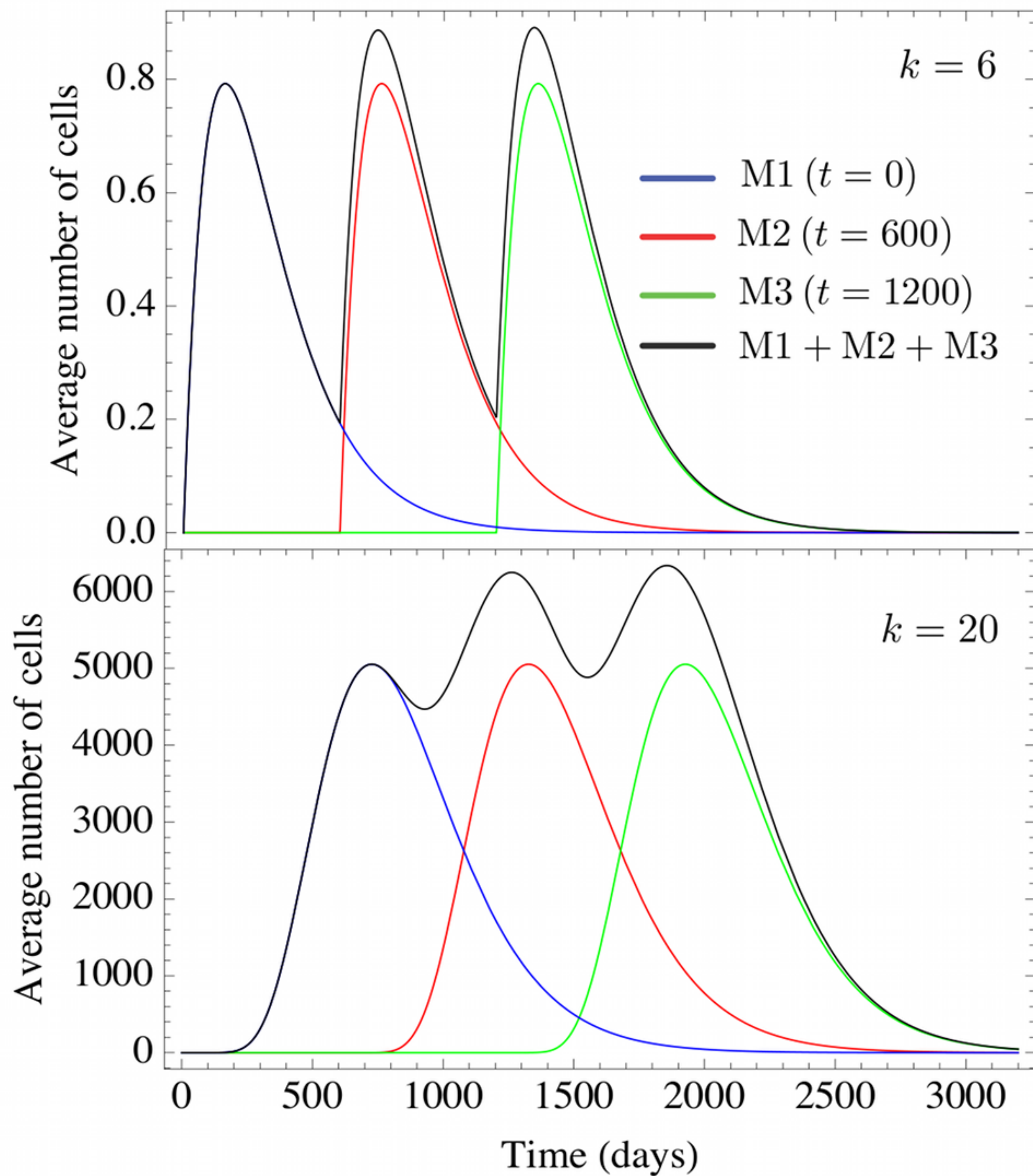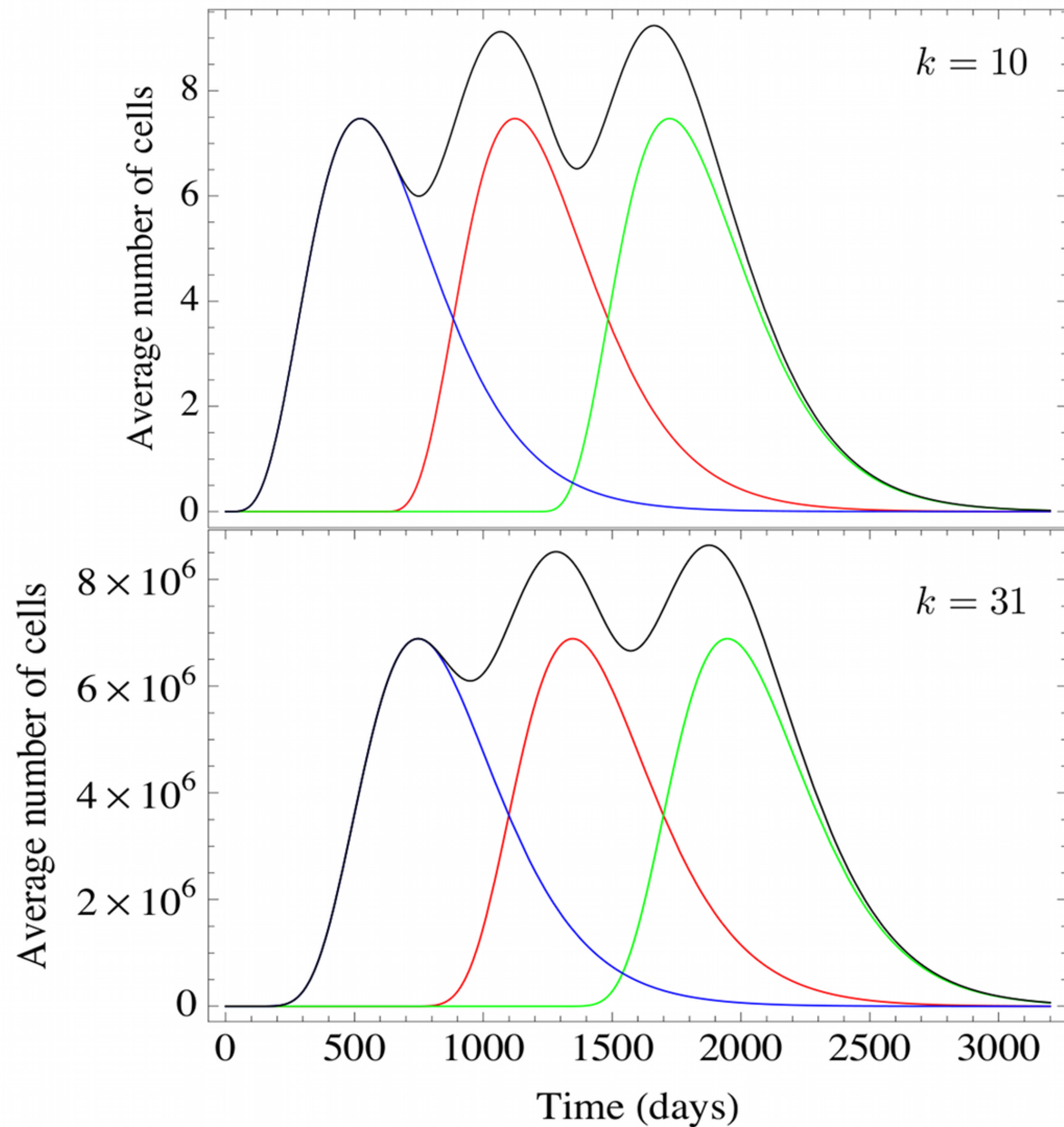

Supplement: Figure S1 — Example of three independent mutation events with equal properties. Shown is the overlapping dynamics of three independent mutation events. (PDF) [file pcbi.1002290.s001.pdf]
