# Supplementary material for: Dynamics of Mutant Cells in Hierarchical Organized Tissues
Source: PLoS Comput Biol. 2011 Dec 1;7(12):e1002290. doi: 10.1371/journal.pcbi.1002290 (PMC3228763; doi:10.1371/journal.pcbi.1002290)
Supplement: Text S1 — Details of mutant cell dynamics. Discussion of mutant cell dynamics and overlapping mutation events. (PDF) [file pcbi.1002290.s002.pdf]

Supporting information for: Dynamics of mutant cells in hierarchical organized tissues

Benjamin Werner, David Dingli, Tom Lenaerts, Jorge M. Pacheco, Arne Traulsen

Extinction, fixation or divergence of mutants

The average cell count in each compartment $\{0, \ldots, k\}$ is given by a system of coupled differential equations

$$\dot{N}_0(t) = 0 \tag{1a}$$

$$\dot{N}_1(t) = -\alpha_1 r_1 N_1 + r_0 N_0 \tag{1b}$$

$$\dot{N}_i(t) = -\alpha_i r_i N_i + 2\varepsilon_{i-1} r_{i-1} N_{i-1} \tag{1c}$$

where r_i is the reproduction rate, ε_i is the differentiation probability and $\alpha_i = 2\varepsilon_i + 2\lambda_i - 1$ is the effective outflux of cells of the i-th compartment, where λ_i is the probability of cell death. Here we want to focus on the long time behavior of mutant cells which occur in a non stem cell compartment j. Thus the initial condition becomes

$$N_i(0) = \begin{cases} 1 & i = j \\ 0 & \text{otherwise} \end{cases}$$

and the system of linear differential equations turns into a homogeneous system

$$\dot{N}_i(t) = \begin{cases} 0 & i < j \\ -\alpha_i r_i N_i & i = j \\ -\alpha_i r_i N_i + 2\varepsilon_{i-1} r_{i-1} N_{i-1} & i > j. \end{cases} \tag{2}$$

The general solution of this linear system is, as discussed in the main text, given by

$$N_i(t) = \begin{cases} 0 & i < j \\ \prod_{l=j}^{i-1} (2 r_l \varepsilon_l) \sum_{h=j}^{i} \frac{(-1)^{i-j}}{R_{hi}^{(j)}} e^{-\alpha_h r_h t} & i \geq j. \end{cases} \tag{3}$$

Our aim is to discuss how this equation depends on the α_h. In our model α_h can range from $-1 \leq \alpha_h \leq 1$. Thus we have three possible cases: (i) In compartment h more cells are lost due to cell death and differentiation than cells are produced due to self renewal. This corresponds to $\alpha_h > 0$. (ii) Loss and gain of cells are equal, this corresponds to $\alpha_h = 0$. (iii) The self renewal outweighs the loss due to cell death and cell differentiation, $\alpha_h < 0$.

Obviously, if all $\alpha_h > 0$, in the long run all exponential functions decline and will reach zero. Thus all mutants in all compartments vanish. This is a stable state.

The second easy case would be $\alpha_k < 0$ for all $k \in \{j, \dots, i\}$. In this case all exponential functions will grow over all limits and the mutant cell count will increase exponential in all compartments.

Next we can think of

$$\alpha_h \begin{cases} < 0 & h = m \\ > 0 & \text{otherwise.} \end{cases} \tag{4}$$

In this case all terms except the m-th in (3) vanish. Thus all mutants in all compartments $j < m$ are washed out, mutants in all compartments $i \geq m$ increase exponentially according to

$$N_{ij}\left(t \rightarrow \infty\right) = \begin{cases} 0 & i < m \\ \frac{(-1)^{i-j}}{\prod_{l=j,l\neq m}^{i}(\alpha_m r_m - \alpha_l r_l)} \prod_{l=j}^{i-1} \left(2 r_l \varepsilon_l\right) e^{-\alpha_m r_m t} & i \geq m \end{cases} \tag{5}$$

All other possible combinations of $\alpha_k \neq 0$ can be constructed using this approach and are simply linear combinations of non vanishing terms.

The most interesting case is

$$\alpha_h \begin{cases} = 0 & h = m \\ > 0 & \text{otherwise.} \end{cases} \tag{6}$$

Mutants in the m-th compartment get stem cell like properties and behave like non stem cells in all other compartments. In this case the general solution (3) becomes

$$N_{ij}\left(t \rightarrow \infty\right) = \begin{cases} 0 & i < m \\ 2^{i-j-1} \frac{r_m}{r_i} \frac{\alpha_m}{\varepsilon_i} \prod_{l=j}^{i} \frac{\varepsilon_l}{\alpha_l} & i \geq m \end{cases} \tag{7}$$

Mutants that occurred downstream of the m-th compartment vanish, in all other compartments the mutant count reaches a fixed value, given by (7). This is a stable fixed point of the system. A small perturbation in N_i will not change the equilibrium. However a small change in α_m leads either to extinction or divergence of the whole mutant cell population.

Overlapping mutation events

The general solutions in the manuscript capture the time dynamics of one mutation event driven or not driven by stem cells. All observed mutant cells are the offspring of one single mutation event. However as a first approximation we assume that mutation events are independent of each other. Further we assume that cells proliferate independent of each other and thus different cell lineages do not interfere. If there are n independent mutation events at times $\{t_1, \dots, t_n\}$, then the total mutant cell count $N_{ij}^{\text{tot}}\left(t\right)$ at time t is a linear combination of all the single lineages $N_{ij}\left(t - t_l\right)$ leading to

$$N_{ij}^{\text{tot}}\left(t\right)=\sum_{l=1}^{n}N_{ij}\left(t-t_{l}\right)H\left(t-t_{l}\right), \tag{8}$$

where $N_{ij}\left(t-t_{l}\right)$ is given by

$$N_{ij}\left(t-t_{l}\right)=\begin{cases}0 & i<j\\ \prod\limits_{l=j}^{i-1}\left(2r_{l}\varepsilon_{l}\right)\sum\limits_{h=j}^{i}\frac{(-1)^{i-j}}{R_{hi}^{(j)}}e^{-\alpha_{h}r_{h}(t-t_{l})} & i\geq j\end{cases} \tag{9}$$

and $H\left(t-t_{l}\right)$ is the Heaviside step function

$$H\left(t-t_{l}\right)=\begin{cases}1 & t\geq t_{l}\\ 0 & \text{otherwise.}\end{cases} \tag{10}$$

An example is provided in figure 1. There one has three independent mutation events at $t_0=0$, $t_1=600$ and $t_2=1200$, where the properties of all three mutations are the same (different properties are possible without any complication). Shown are the mutant cell counts in the downstream compartments $\{6,10,20,31\}$. In vivo measurements can typically not distinguish if mutants are caused by a single mutation event or are caused by independent events. Thus one would obtain on average the black line of compartment 31, which is caused by three independent events originally. It is likely that mutations occur more often in downstream compartments but they never reach a high cell count. However one can expect to find an almost constant but small count of certain mutations in the blood stream that are caused by many independent mutation events.

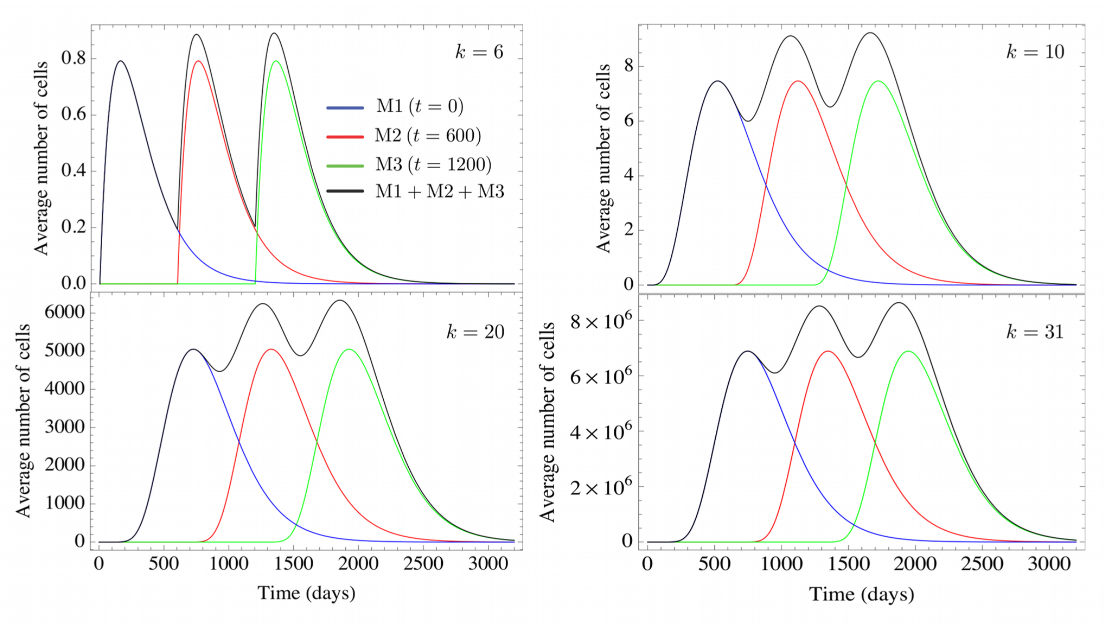

Figure 1: **Example of three independent mutation events with equal properties.** In this case mutations occur in the compartment $j = 5$ at times $t_1 = 0$, $t_2 = 600$ and $t_3 = 1200$. Shown are the numbers of the total mutant count (black line) and the single lineages (blue, red and green line) in the compartments $i = \{6, 10, 20, 31\}$ in a model for hematopoiesis as in the main manuscript.
